# The representation of patient experience and satisfaction in physician rating sites. A criteria-based analysis of English- and German-language sites

**DOI:** 10.1186/1472-6963-10-332

**Published:** 2010-12-07

**Authors:** Swantje Reimann, Daniel Strech

**Affiliations:** 1 Hannover Medical School, CELLS - Centre for Ethics and Law in the Life Sciences, Institute for History, Ethics and Philosophy of Medicine, Carl-Neuberg-Str. 1, 30625 Hannover, Germany

## Abstract

**Background:**

Information on patient experience and satisfaction with individual physicians could play an important role for performance measures, improved health care and health literacy. Physician rating sites (PRSs) bear the potential to be a widely available source for this kind of information. However, patient experience and satisfaction are complex constructs operationalized by multiple dimensions. The way in which PRSs allow users to express and rate patient experience and satisfaction could likely influence the image of doctors in society and the self-understanding of both doctors and patients. This study examines the extent to which PRSs currently represent the constructs of patient experience and satisfaction.

**Methods:**

First, a systematic review of research instruments for measuring patient experience and satisfaction was conducted. The content of these instruments was analyzed qualitatively to create a comprehensive set of dimensions for patient experience and patient satisfaction. Second, PRSs were searched for systematically in English-language and German-language search engines of Google and Yahoo. Finally, we classified every structured question asked by the different PRS using the set of dimensions of patient experience and satisfaction.

**Results:**

The qualitative content analysis of the measurement instruments produced 13 dimensions of patient experience and satisfaction. We identified a total of 21 PRSs. No PRSs represented all 13 dimensions of patient satisfaction and experience with its structured questions. The 3 most trafficked English-language PRS represent between 5 and 6 dimensions and the 3 most trafficked German language PRSs between 8 and 11 dimensions The dimensions for patient experience and satisfaction most frequently represented in PRSs included diversely operationalized ones such as *professional competence *and *doctor-patient relationship/support*. However, other less complex but nevertheless important dimensions such as *communication skills *and *information/advice *were rarely represented, especially in English-language PRSs.

**Conclusions:**

Concerning the potential impact of PRSs on health systems, further research is needed to show which of the current operationalizations of patient experience and satisfaction presented in our study are establishing themselves in PRSs. Independently of this factual development, the question also arises whether and to what extent health policy can and should influence the operationalization of patient experience and satisfaction in PRSs. Here, the challenge would be to produce a set of dimensions capable of consensus from among the wide range of operationalizations found by this study.

## Background

For questions about health and sickness in general, a large share of the information relevant to decision-making is publicly available through digital media (particularly the internet). Anyone can learn about the characteristics of certain illnesses (cause, symptoms, diagnostic criteria) and of the available medical measures (effectiveness, dosage, side effects) (e.g. [1,2]). This information is more practically relevant if it facilitates the primary goals and ethical principles of medicine [3,4]: (i) the welfare of patients, (ii) the respect for and promotion of patient autonomy and health literacy, and (iii) social justice.

Alongside information on illnesses and medical measures, it could also be relevant to the primary goals of medicine to acknowledge patients' satisfaction with physicians and provide information on this [5-7]. However, health services research has recently criticised the concept of patient satisfaction because of its inherent sources of bias [8]. Patients, for example, can describe high levels of satisfaction at the same time as describing experiences that are suboptimal. Thus in light of the limitations of patient satisfaction research, a recent trend in health services research has favored questions about patients' experiences [8]. Despite the great amount of active research and scientific publications in the field of patients' experience and satisfaction [7-9] there is only very little publicly available information on patients' experience and satisfaction concerning *individual *doctors or hospitals [10].

Physician rating sites (PRSs) are a new web-based source for peer-to-peer information on individual physicians. PRSs provide information about a physician's address, opening hours and certifications [10]. Next to this factual information, a major objective of PRSs is to collect and present information about patients' experience and satisfaction with individual physicians and their practices. PRSs could, therefore, improve informed provider choice [11,12] and are similar to other peer-to-peer consumer information websites that allow the rating and discussion of the experience and satisfaction with hotels, restaurants or technological devices. In contrast to the rather expert-driven approaches to information on patients' experience and satisfaction, such as the CAHPS Clinician and Group Survey [7], PRSs are a consumer-driven alternative.

So far, very little research has been done on PRSs [10,13]. In contrast to this lack of research, there is controversial discussion about the impact of PRSs in several health systems. The British National Health Service (NHS) and the leading sickness fund within the German statutory health insurance have encouraged patients to review their physicians and hospitals [14,15]. American and German physician organizations, including the American Medical Association (AMA) and the German Medical Association (BÄK), have been rather opposed to the development of PRSs, arguing, for example, that the identity of patients cannot be confirmed, reviews will be excessively negative, and physicians' responses will be hampered by confidentiality issues [16]. To our knowledge, patient organizations were rather silent about their views on positive and negative consequences of PRSs.

Besides the technical and judicial questions surrounding PRSs, future discussion and decision making on PRSs also have to consider the content and rating options of PRSs. The practical relevance of information on patient experience and satisfaction - with respect to the achievement of the primary goals of medicine - depends significantly on how the complex construct of patient experience and satisfaction is represented in PRSs.

Our study examines, qualitatively and quantitatively, the extent to which PRSs currently represent patients' experiences and the construct of patient satisfaction as measured by research instruments.

## Method

### The framework of dimensions representing patients' experience and satisfaction

An examination of the extent to which PRSs represent patients' experience and the construct of patient satisfaction requires a comprehensive catalogue of dimensions. Given the variety of instruments for measuring patients' experiences and satisfaction, which to some extent vary significantly in their exclusion and inclusion of individual dimensions, we decided to compile one single framework of dimensions on the basis of a systematic review of reviews of relevant measurement instruments. We conducted a systematic search in MEDLINE using the following search algorithm: ("Health Surveys"[Mesh] OR "Questionnaires"[Mesh]) AND ("Patient Satisfaction"[Mesh] OR "Patient Experience") AND "Review "[Publication Type]. We included all reviews that reviewed instruments for measuring patients' experience and satisfaction with resident or clinical doctors in general. We obtained the original versions of the potentially relevant measurement instruments mentioned in the reviews. We included all measurement instruments that (i) provided at least one dimension to measure patients' experience or satisfaction in general and (ii) reported information on reliability (re-test reliability, internal consistency) and validity. Measurement instruments on illness- or setting-specific patient satisfaction were excluded, for example [17,18]. All bibliographies of the reviews and measurement instruments we included were examined for further relevant measurement instruments. We extracted dimensions for patients' experience and satisfaction from the instruments we included through a qualitative content analysis [19]. After an initial reading, both authors individually and jointly organized all mentioned dimensions into conceptual relationships and higher order dimensions. After discussion both authors were able to reach a consensus about all discrepancies in reviewing and analyzing the instruments. The resulting set of 13 dimensions arranged into three subgroups produced the framework for the analysis of the PRSs.

### Systematic search for physician rating sites

In November 2009 we did a systematic search for PRSs in the English-language search engines of Google and Yahoo with the following search terms: "doctor rating sites", "physician rating sites", "rate a physician", "rate a doctor", "search a doctor", "find a doctor". In the German-language search engines of Google and Yahoo we used the terms: "Arztbewertung", "Ärzte finden", "Ärzte bewerten", "Arztsuche". We checked the first 100 hits of every search. All websites were labeled PRSs and included that allowed the specific qualitative (narrative) or quantitative expression of patients' experiences or satisfaction with physicians in a structured format. We restricted our search to the first 100 hits of every search because it is to be assumed that the PRSs identified in this way are the sites that a potential user would run into in his or her search, and thus represent the most frequently used sites. Website traffic data was estimated using Alexa (see table 1) http://www.alexa.com/siteinfo.

**Table 1:** Operators and ranking of websites

Physician Rating Sites	Operator/Sponsor	Traffic ranking#		Sites linking in
			
		Global	Country specific	
1 Vitals.com	Owned by MDx Medical, Inc with headquarters in Lyndhurst, NJ. Comprised of a team of dedicated experts in the fields of healthcare, marketing, and database management.	9,656	2,044*	381

2 RateMDs.com	Just average patients who happen to know how to make websites; not affiliated with any medical organizations.	27,042	8,952*	494

3 Vimo.com	Founded by Internet industry veterans from WebMD and Valicert; funded by Bessemer Venture Partners, Trinity Ventures, and Partech International.	104,825	32,695*	427

4 Drscore.com	Operated by the Medical Quality Enhancement Corp., founded by Steven R. Feldman, M.D., Ph.D.	132,510	45,120*	129

5 CheckMD.com	No information on owner or sponsor found	155,051	37,745*	92

6 Mydochub.com	No information on owner or sponsor found	444,885	394,003*	121

7 Doctortree.org	DoctorTree.org; Encino, CA	493,089	119,259*	95

8 Bookofdoctors.com	No information on owner or sponsor found	551,810	117,124*	61

9 Findadoc.com	Team of doctors and programmers who created FindaDoc to make the daunting and sometimes frustrating process of finding the best doctor as simple as possible	697,002	221,478*	157

10 Healthcarereviews.com	Owner and operator: Women's College Hospital	1,057,783	296,088*	58

11 Drscorecard.com	Built and operated by people that are free from ties to medical businesses, medical associations, hospitals, clinics, doctors, and even free from patient advocacy groups.	n.a.	n.a.	n.a.

12 Ratemymd.ca	No information on owner or sponsor found	2,267,175	82,274**	5

13 Imedo.de	imedo GmbH, Berlin	21,709	1,187***	999

14 Jameda.de	jameda GmbH, München; Tomorrow FOCUS AG and FOCUS Magazine Publisher	31,313	1,765***	410

15 Docinsider.de	Andingo Capital GmbH, Berlin; Funding by the German Ministry for Education and Research (BMBF) and the German Ministry for Economy and Technology (BMWi)	63,992	3,311***	301

16 Esando.de	Comventure GmbH, Ludwigshafen	99,668	5,624***	162

17 Medführer.de	medführer Online-publisher, GmbH in cooperation with University of Trier	114,165	6,029***	267

18 Helpster.de	Helpster GmbH, Munich	169,818	10,225***	128

19 Topmedic.de	ArztData GmbH, Hamburg	1,423,039	94,349***	46

20 Die-arztempfehlung.com	Endverbraucher Ltd. & Co., Fürth	n.a.	n.a.	n.a.

21 Mein-guter-arzt.de	Mediaseed GmbH, Stuttgart	n.a.	n.a.	30

#Alexa Traffic Rank: The rank is calculated using a combination of average daily visitors to website and pageviews on this website over the past 3 months. Sites Linking In: The number of sites linking to specific website. Multiple links from the same site are only counted once. (http://www.alexa.com; 01.07.2010)

* Country: USA/** Country: Canada/*** Country: Germany/n.a. = not available, GmbH = limited liability company

### Structured content analysis of physician rating sites

We used the 13 framework dimensions to classify each rating option or structured question provided by the different PRSs. Multiple classifications for one rating option were possible.

## Results

The MEDLINE search yielded 401 references from which 55 were reviews of instruments for assessing patients' experience or satisfaction. From these we identified 20 English-language or German-language instruments (see table 2) with sufficient reliability and validity (range of test reliability: 0.62 [20] - 0.94 [21]); range of test validity: 0.54 [22] - 0.98 [23]) [24]. The qualitative content analysis of the 20 measurement instruments produced 13 dimensions of patients' experience and satisfaction (see tables 3, 4, and 5).

**Table 2:** Instruments for assessing patients' experience and satisfaction

Instrument	Author
ABIM-10, American Board of Internal Medicine	Webster [34]

CSQ, Consultation Satisfaction Questionnaire	Baker [35]

CSS, Consumer Satisfaction Survey	Defossez [21]

"dialogue", consultation satisfaction questionnaire	National Clinical Audit Centre [36]

EUROPEP	Klingenberg [37]

GPAS, General Practice Assessment Survey	National Primary Care Research a. Development Centre [20]

IPQ, Improving Practice Questionnaire	Greco [38]

KPF-A	Brinkmann [39]

KPF, Kölner Patientenfragebogen	Pfaff [40]

McKinley et al.	McKinley [22]

MISS-21, Medical Interview Satisfaction Scale	Meakin [41]

OPEQ, Outpatient Experiences Questionnaire	Garratt [42]

PDRQ-9, Patient-Doctor-Relationship Questionnaire	Van der Feltz-Cornelis [43]

PEQ, Patient Experience Questionnaire	Steine [23]

PSQ, Patient Satisfaction Questionnaire	Grogan [44]

QP-Qualitätspraxen	Nubling [45]

Qualiskope-A	Gericke [46]

REPERES-60, Recherche Evaluative sur la Performance des Réseaux de Santé	Defossez [21]

SOSQ, Seattle Outpatient Satisfaction Questionnaire	Fan [25]

ZAP - Zufriedenheit in der ambulanten Versorgung - Qualität aus Patientensicht	Bitzer [47]

**Table 3:** Characteristics of encounter between doctor and patient

Dimensions --------------------- PRS	Doctor-patient-relationship and Support	Communication skills	Trust	Professional care	Information and advice
1 Vitals.com	Bedside manner (caring)/Spending enough time with me			Accuracy in diagnosing a problem/following up as needed after my visit	

2 RateMDs.com	Helpfulness (Is the doctor approachable and nice? Is he rude, arrogant, or just plain mean? Does he have a good bed-side manner?)			Knowledge	

3 vimo.com	Personal skills: Friendly and approachable	Takes time to answer questions. (Do you feel rushed while talking? Does the doctor show concern for you? Do you have to repeat your case history every time?		Knowledge and skill: Diagnoses problems accurately, recommends best treatment. (Does your doctor have up-to-date medical skills? Can you get recommendation to a specialist easily?)	

4 Drscore.com	friendliness and caring attitude/The extent that the doctor includes you in decisions about your care and treatment/How well MD follows-up on any problems or concerns you have			thoroughness of exam or check-up/Ability to get all of the care for your health problem or illness at this clinic.	How well all questions were answered/instructions on how to take care of your illness or health condition

5 checkMD.com	Friendly			Competence	Informative

6 mydochub.com	Personal skills	Listening to you		Knowledge and professionalism	

7 doctortree.org	Bedside manner (Do they make you feel comfortable)			Knowledge of medicine	

8 Bookofdoctors.com	Personal attention during visit/shows caring & compassion	Ability to communicate		Willingness to make referrals/quality of referrals	Explanation/Coordination of medications

9 Findadoc.com	Bedside manner (attitude and conduct of a physician in the presence of a patient)		Patient confidence	Medical knowledge (how knowledgeable the doctor is in his or her field)	

10 healthcarereviews.com	Helpful			Knowledgeable	

11 Drscorecard.com					

12 Ratemymd.ca	Caring, Helpfulness	Understanding		Medical knowledge	

13 Imedo.de			Privacy - upholding the need for a confidential and private atmosphere	Interpersonal factors - was the doctor competent in your estimation	Interpersonal actors - did you feel well-advised by the doctor? (Information on preventative exams, preventative options, diagnosis, prognosis, therapy, written information, education)

14 Jameda.de	Friendliness (the doctor's conduct, openness, was the doctor empathetic and considerate)	Did the doctor listen to you enough? Were your questions answered thoroughly and patiently?	Relation of trust - did you feel yourself in good hands with this doctor?	Was the doctor's diagnosis later confirmed? Did he follow up with the appropriate treatment?	Education about illness and treatment/education about illness and treatment (were the explanations understandable to you?)

15 Docinsider.de	Satisfaction with the understanding, empathy, humaneness, being taken seriously, treated as a person; being involved in the decisions (satisfaction with support and advice)		Do you trust this care professional?	Satisfaction with professional competence (thorough and diligent medical treatment, readiness to refer to other specialists)	Satisfaction with information and advice

16 Esando.de	Doctor's friendliness, timeframe			Type and extent of examination	Doctor's explanations, explanation of the results

17 Medführer.de	Doctor-patient relation (did the doctor take enough time; how understandable were your instructions after leaving the office)		Medical secretary (satisfaction with the atmosphere of privacy)	Medical services (satisfaction with the professional abilities of the doctor)	Medical services (satisfaction with the information and advice, involvement in the treatment)

18 Helpster.de					

19 Topmedic.de	Doctor (patient involvement in choice of alternative therapies, friendliness, readiness to help, respectfulness)		Doctor (maintenance of the atmosphere of privacy)	Doctor (thoroughness of examination, professional competence)	Doctor (comprehensibility of explanation)

20 Die-arztempfehlung.com				I find the relation between my treatment and the ensuing costs to be very good	All of my questions were answered by the doctor and his/her team to my satisfaction

21 Mein-guter-arzt.de					Advice

**Table 4:** Organizational aspects of medical practice

Dimensions ------------------ PRS	Medical and technical facilities	Accessability/Availability	Office characteristics	Office organization, Waiting time	Office staff
1 Vitals.com		Ease in getting an appointment		Waiting time during a visit	Courtesy and professionalism of office staff

2 RateMDs.com				How long does the doctor keep you waiting?	How is the service and helpfulness of the doctor's staff?

3 vimo.com		Availability - Appointments are available easily and on a timely basis. (Is there good after-hours support? Can the doctor be reached over phone? How about availability by email?)		Punctuality - Is reasonably punctual with appointments. (How is the wait time in the office? Has the doctor cancelled appointments?)	Office Staff - Office staff is courteous and professional. Do your phone messages get communicated to the doctor? Is the staff sensitive to you and your condition?

4 Drscore.com	Ability to get all of the care for your health problem or illness at this clinic.	The ability to see the health care provider you wanted to see at this clinic/Getting the advice or help you needed after office hours.	Patient convenience such as ample parking and location of office	Getting your test results back in a timely manner./About how many days did you have to wait to get an appointment to see the doctor?/After arriving at the office, how many minutes did you wait before seeing the doctor/About how many minutes did the doctor spend with you in your most recent visit	The friendliness and courtesy of the office staff.

5 checkMD.com				Timely	Office Staff

6 mydochub.com				Punctuality/approximate time spent in waiting room	Office staff

7 doctortree.org				Time spent in waiting room	

8 Bookofdoctors.com		Doctor availability	Cleanliness of office	Waiting room time/Returns calls in a timely fashion/Accuracy of billing	Professionalism of staff

9 Findadoc.com			Office Setting (This is a measurement of the appearance and cleanliness, of a doctor's facilities)	Wait Time (How long you have to wait to get an appointment and how long you have to wait in the doctor's office.)	Office Staff (How well the office staff meets your needs as a patient)

10 healthcarereviews.com				Wait Times/Costs/Fees	

11 Drscorecard.com	Medical equipment	Time you waited to get an appointment after requesting one		Cost/Time you waited in the waiting room	The Office staff/The nurses

12 Ratemymd.ca				Punctuality, post appointment action	The secretary/administrative assistant

13 Imedo.de	Office facilities: no computers	Availability of appointments: how quickly did you get an appointment?	Office organization, information on the organizational structure of the office	Punctuality, how long you had to wait in the waiting room	Staff, how friendly and well-organized were the office staff in making appointments and in the office

14 Jameda.de		Waiting times for an appointment (optional), availability by telephone (optional), public accessibility, parking spaces (optional)	Office facilities (optional); Entertainment in the waiting room (optional)	Waiting times in the office (optional)	Attentiveness and friendliness of the entire personnel (optional)

15 Docinsider.de		Satisfaction with the waiting time for an appointment, how long did you wait?	Satisfaction with the atmosphere	Satisfaction with the waiting times, how long did you wait; house visits	Satisfaction with the friendliness of the office staff

16 Esando.de	Office facilities (up-to-date)	Office organization (making appointments)	How did you find the office facilities (well-kept waiting room, tidy examination room)	Office organization (office waiting time, attended to during the waiting time)	

17 Medführer.de		Appointment (were you satisfied with the time needed to get an appointment with this doctor), getting there (parking spaces, directions, accessibility), doctor-patient relation (is the doctor available by telephone)	Reception (satisfaction with the diversions in the office); Office premises (service, ambiance, cleanliness and hygiene)	Reception (satisfaction with the organization and waiting times); Medical secretary (access to patient files)	Reception (friendly greeting); Medical secretary (professional competence and friendliness of the team)

18 Helpster.de			Personal assessment (how satisfied were you with how you were treated - thoroughness, cleanliness)	Personal assessment (how satisfied were you with the waiting times)	Personal assessment (how satisfied were you with the friendliness)

19 Topmedic.de		Organisation and service (availability by telephone, available office hours, availability of appointment, accessibility with public transportation, parking)	Appearance (equipment, hygiene, signs, clear arrangement)	Organization and service (waiting time in the office)	Personnel (friendliness, readiness to help, respetfulness, clarity of information, preservation of privacy)

20 Die-arztempfehlung.com		The appointments were always kept	The premises and the facilities of the office left a very good impression on me	There were hardly waiting times	I was always treated with friendliness and consideration by the office staff

21 Mein-guter-arzt.de			Atmosphere, equipment	Waiting time	

**Table 5:** Overarching assessment categories

Dimensions ----------------------------- PRS	Success of outcome	General satisfaction	Willingness to recommend the doctor
1 Vitals.com		Overall, what is your opinion of this doctor?	

2 RateMDs.com	How did his treatments work for you?		

3 vimo.com			

4 Drscore.com	Your treatment success	Overall rating	

5 checkMD.com			

6 mydochub.com		Your patient satisfaction score rating for this visit:	

7 doctortree.org			

8 Bookofdoctors.com		Overall quality of care	Would you recommend this doctor?

9 Find a doc.com		Patient satisfaction (This is your overall level of satisfaction with the doctor and his or her treatment)	recommend this doctor to a family member or close friend

10 healthcarereview.com	Helpful	Overall rating	

11 Drscorecard.com		How would you rate this doctor and the office overall?	

12 Ratemymd.ca	Helpfulness		

13 Imedo.de			

14 Jameda.de	Were you better after the treatment?	Satisfaction with the treatment	

15 Docinsider.de	Assessment of the quality of treatment in general	General satisfaction	Would you recommend this doctor?

16 Esando.de		Total impression	Readiness to recommend the doctor

17 Medführer.de	Medical services (satisfaction with the results of the treatment)		

18 Helpster.de	Medical assessment (How do you subjectively judge the success of the treatment?)	Total assessment	

19 Topmedic.de		Total rating	Recommendation (to your best friend)

20 Die-arztempfehlung.com	I find the result of the treatment to be very good		I would feel very good recommending this doctor to friends and relatives

21 Mein-guter-arzt.de		Total impression	

A total of 12 English-language and 9 German-language PRSs were identified (see tables 3, 4, and 5) [last checked in April 2010]. No PRS represented all 13 dimensions of patients' experience and satisfaction with its structured questions. The number of dimensions for patients' experience and satisfaction represented in PRSs range from 3 (checkMD.com, doctortree.org) to 11 (Jameda.de, Docinsider.de). For data about the traffic of PRSs see table 1. While the 3 most trafficked English-language sites (vitals.com, RateMD.com, vimo.com) only represent between 5 and 6 dimensions (see tables 3, 4, and 5) the 3 most trafficked German-language sites (Imedo.de, Jameda.de, Docinsider.de) represent between 8 and 11 dimensions.

Among the dimensions most frequently represented in PRSs are the less complex dimensions such as *office organization/waiting time *(in 21 PRSs) and *office staff *(in 17 of 21 PRSs). On the other hand quite complex and thus diversely operationalized dimensions of patient experience and satisfaction such as *professional competence *(in 18 of 21 PRSs) and *doctor-patient relationship *(in 16 of 21 PRSs) are also often found on these sites. However, other dimensions that allow users to rate important aspects of the encounter between doctor and patient were rarely represented. For example, only 4 of 12 English and 1 of 9 German PRSs provided structured questions about physicians' *communication skills*. Also only 3 of 12 English PRSs but 8 of 9 German PRSs provided structured questions concerning how well physicians offer *information and advice*.

The following sections show how the dimensions of patients' experience and satisfaction are represented both by the research instruments and in the PRSs. We will begin by qualitatively describing the operationalization of each dimension by the research instruments. This operationalization formed the basis for the classification of rating options in the PRSs that we will present subsequently. To facilitate reading we separated the 13 dimensions of patients' experience and satisfaction into 3 subgroups (see tables 3, 4, and 5) that aim to group together (i) characteristics of the encounter between doctor and patient (e.g. doctor-patient relationship, communication) in table 3 (ii) organizational aspects of the medical practice (e.g. equipment, personnel, organization) in table 4 and (iii) overarching assessment categories (e.g. general satisfaction) in table 5. Numbers in parentheses indicate the corresponding PRSs specified in tables 3, 4 and 5.

### Characteristics of the encounter between doctor and patient

#### Doctor-patient relationship

*Patients' experience and satisfaction research instruments include *the personal skills of the doctor such as patience, taking the patient seriously, being friendly, caring, trustworthy, diligent, empathetic and humane. Additional characteristics, such as seeing the patient as an equal partner or as a person and not as a number, are often classified under patient-centeredness. Additionally the quality of the doctor-patient relationship has been operationalized by means of doctor behavior such as showing interest in the patient, maintaining a respectful manner, and allowing the patient to ask necessary questions as well as supporting the patient through his or her mental, social and bureaucratic difficulties.

*PRS*: 16 PRSs (76%) allow for an explicit assessment of certain aspects of the doctor-patient relation as described above (1-10, 12, 14-17, 19). Three English-language PRSs use the term "bedside manner" as a catch-all term for a particular behavior on the part of the doctor, but explain it differently - one more descriptively as the "attitude and conduct of a physician in the presence of a patient" (9) and another in clear normative terms as "do they (physicians) make you feel comfortable" (7). Other criteria that we correlated with the dimension of doctor-patient relation were helpfulness (2, 10, 12), approachability and friendliness (2-5, 14-16, 19), spending enough time with the patient (1, 17), shows caring or concern (8, 12), and shows compassion (8).

#### Communication skills

*Patients' experience and satisfaction research instruments *include the ability to listen, to communicate diagnoses to the patient appropriately, to inform the patient about decisions and to (transparently) involve the patient in the process of examination and treatment. Even if there is some concrete overlap between the dimensions of communication and doctor-patient relation, we find it possible and productive to maintain a distinction. The Association of American Medical Colleges (AAMC) also emphasizes this distinction between communication skills and interpersonal skills [25], whereby the communicative skills include above all effective listening, appropriate questioning and the provision of information. Interpersonal qualities are seen more in the establishing of a trusting relation [26].

*PRS*: Five sites (24%) asked explicitly about this dimension as the "ability to communicate" (8), "understanding" (12), or "listening to you" (6). The criteria of "takes time to answer questions", "feel rushed while talking" (3) and the question of sufficient conversational time (14) also represent the doctor's communicative skills.

#### Trust

*Patients' experience and satisfaction research instruments include *questions about whether the patient feels trust in the particular doctor, whether an atmosphere of privacy obtains or whether the patient can open up to entrust the doctor with all of the necessary information.

*PRS*: Six sites (29%) ask about this dimension. The explanations hardly differ at all; they usually concern the preservation of the sense of privacy (13, 17, 19) and then ask quite unspecifically about whether the patient trusts the particular doctor, for example with the question of whether one feels oneself in good hands (9, 14) or "Do you trust this care professional?" (15).

#### Professional competence

*Patients' experience and satisfaction research instruments include *the doctors' knowledge of his or her own (professional) limitations (e.g. prompt referral, collaboration with other doctors, admitting his or her own errors) and the aspect of diligence (e.g. investigating all possible causes, correct diagnosis, thorough examination, responsibility). This dimension also includes knowledge of all aspects of the medical therapy and the appropriateness of the treatment (e.g. medications without or with only minor side effects, no excess treatment, individualized medical treatment, no doubled examinations, awareness of price).

*PRS*: 18 sites (86%) ask for an assessment of this dimension (1-10, 12-17, 19, 20). The questions range from unspecified aspects such as "knowledgeable" or "Knowledge and Professionalism" (2, 5-7, 9, 10, 12, 17, 19) to more comprehensive descriptions of professional competence such as "Was the doctor's diagnosis later confirmed? Did he follow up with the appropriate treatment?" (14) or "Does your doctor have up-to-date medical skills? Can you get a recommendation to a specialist easily?" (3).

#### Information and advice

*Patients' experience and satisfaction research instruments include *the content and scope of what is communicated. Hence this dimension particularly concerns how well and how transparently the patient is informed during the medical treatment. This includes the information given (e.g. understandable information about the causes and process of the illness and the side effects of diagnosis and treatment) and advice (e.g. information on self-help groups or nutritional advice).

*PRS*: Eleven sites (52%) covered this dimension in their assessment (4, 5, 8, 13-17, 19-21). Two of the sites only named the dimension as "informative" (5) and "advice" (21), whereas the other assessment templates provided the user with more thorough explanations, for example "Information on preventative examinations, preventative options, diagnosis, therapy, written information, education" (13) or "All of my questions were answered by the doctor and his or her team to my complete satisfaction. I felt that I was advised very well." (20).

### Organizational aspects of the medical practice

#### Medical and technical facilities

*Patients' experience and satisfaction research instruments include *the technical facilities of the physician's practice (or the hospital).

*PRS*: Four of the sites (19%) include this dimension in the assessment (4, 11, 13, 16). This dimension is represented by questions such as "How did you find the technical equipment of the medical practice (technically up-to-date)?" (16) or "Ability to get all of the care for your health problem or illness at this clinic" (4).

#### Accessibility/availability

*Patients' experience and satisfaction research instruments include *the doctor's availability by telephone outside of visiting hours, the accessibility of the office or clinic (e.g. wheel-chair accessible, directions by public transportation are available, parking nearby, etc.) as well as the possibility of house visits. An acceptable waiting period for an appointment and an arrangement of appointments suitable to the patient are also included.

*PRS*: More than half of the sites (12; 57%) asked about aspects of accessibility/availability (1, 3, 4, 8, 11, 13-17, 19, 20). Often only one representative criterion was named such as "availability" or "arrangement of appointments" (1, 8, 16, 20). More concrete questions concerned the possibility of house visits, the waiting time for an appointment, the possibility of reaching the doctor by telephone outside of visiting hours or per email, the allocation of appointments, and available parking.

#### Office characteristics

*Patients' experience and satisfaction research instruments include *the atmosphere in the office or clinic (team spirit, attractive waiting room, cleanliness), separate changing rooms, or play-areas for children.

*PRS*: This dimension was included by 12 sites (57%). It was represented by "cleanliness of the office" (8), "entertainment in the waiting room" (14) and "office setting" (9) or more specifically by the hygiene or atmosphere of the office or the parking situation (4, 13, 15-21).

#### Office organization

*Patients' experience and satisfaction research instruments include *time management (brief waiting periods within the office, good office organization), customer-orientation (emphasis on the patient, no interrupting phone calls, equal service for all patients, shorter waiting times for emergencies) and service (priority for mothers with children, referral given on the same day, copies of all test results).

*PRS*: This dimension is included in all of the rating sites. There are hardly any differences in the weight given individual aspects of office organization: waiting times and promptness are the primary concerns. One site goes into this point in greater detail: "Getting your test results back in a timely manner./After arriving at the office, how many minutes did you wait before seeing the doctor?/About how many minutes did the doctor spend with you in your most recent visit?" (4). On English-language websites this category also sometimes includes an estimate of the costs of the treatment (8-11).

#### Office staff

*Patients' experience and satisfaction research instruments include *the competence and friendliness of the staff as well as their interaction within the team.

*PRS*: 17 of the sites (81%) cover this aspect. Several sites ask about this without any further explanation, particularly concerning the friendliness, helpfulness and professionalness of the staff (1-6, 8, 11-15, 17-20). Four sites provide examples of how the friendliness of the staff could be demonstrated, e.g.: "How friendly and well-organized were the office personnel in the office and in making appointments" (13), "Base your opinion on your experience making an appointment, how you are greeted for an office visit, and how well the office staff meets your needs as a patient" (9).

### Overarching assessment categories

#### Success of outcome

*Patients' experience and satisfaction research instruments include *assessments of effectiveness (e.g. help by the doctor, effective medications), the suitability of the therapy relative to the diagnosis, the disappearance of symptoms, the amelioration of the complaint, and the increase in functional ability and quality of life.

*PRS*: Nine of the sites (43%) ask explicitly about the success of the treatment, for example with questions such as: "Were you better after the treatment?" (14), "How would you subjectively assess the success of the treatment?" (18), or "How did his treatments work for you?" (2). All 9 PRSs (2, 4, 10, 12, 14, 15, 17, 18, 20) assess this dimension by asking about the subjective treatment success; there are no detailed descriptions of the reduction of specific symptoms, ability to go back to work, increase in quality of life, etc.

#### General satisfaction

*Patients' experience and satisfaction research instruments include *the aggregate assessment as a general summary of all previous assessments.

*PRS*: 13 of the sites asked about general satisfaction (62%), for example using terms such as "overall satisfaction" (1, 6, 8, 9, 14, 15) or by asking for a total rating/assessment (4, 10, 11, 16, 18, 19, 21). No site said anything in greater detail about this total assessment. The visual presentation of the assessment of a doctor (ranging from check-marks to stars, plus points, and other symbols) or the option of writing free commentary might implicitly represent general satisfaction. On this broader definition the dimension *general satisfaction *would be represented on all sites.

#### Willingness to recommend the doctor

*Patients' experience and satisfaction research instruments include *patients' willingness to recommend a doctor and generally describe this dimension as: would the patient be able to recommend this doctor (or this clinic) to friends and relatives?

*PRS*: Only six sites (29%) explicitly posed this question to their evaluating users (8, 9, 15, 16, 19, 20). This dimension is similar to the representation of general satisfaction: one would expect a high level of satisfaction to correspond with a high willingness to recommend the doctor, especially as this is precisely the point of the sites: to allow people to share their experiences with those looking for a suitable doctor and to offer their own experiences to others who might find it useful and helpful.

#### Possibility of free commentary

86% of the sites (18) allow for users to write free commentary (except 5, 13, 17). One site only offers assessment by commentary without indicating or asking about any other dimensions, and was thus excluded from the criteria-based analysis. "The operators of this site assume that "rating" doctors doesn't give you the kind of information you need. [...] You can't make a "best fit" healthcare choice when all you have is a number between 0 and four" ("Our Philosophy" [27]).

## Discussion

The 21 PRSs examined here show a clearly heterogeneous representation of the different dimensions of patient experience and satisfaction as viewed from a quantitative and qualitative perspective. Our quantitative findings show that the most trafficked English-language PRSs currently only represent between 5 and 6 of the 13 dimensions that can play a role in assessing patient experience and satisfaction. However, the most trafficked German-language PRSs represent between 8 and 11 of the 13 dimensions.

Findings from the qualitative analysis demonstrate that the most frequently represented dimensions in PRSs include diversely operationalized ones such as *professional competence *and *doctor-patient relationship*. To assess professional competence, for example, PRSs provide heterogeneous questions such as: "Does your doctor have up-to-date medical skills", "Was the doctor's diagnosis later confirmed? Did he follow up with the appropriate treatment?", "Thoroughness of examination" and others..

The PRSs identified using our search dimensions are the sites that a potential user would run into in his or her search. Nevertheless, we cannot exclude the possibility of other PRSs not discovered by our search algorithm. However, we do not aim at completeness; instead we interpret the sites examined here as a faithful representation of existing sites and assume that other sites not examined will deviate minimally.

We only examined the structured questions asked by the PRSs and not the actual patient reviews. Therefore we did not include the content of open and narrative feedback sections which may represent additional specifications of the 13 dimensions for patients' experience and satisfaction.

At the current state of research, we cannot give evidence-based recommendations about which and how many dimensions of patients' experience and satisfaction need to be represented in PRSs to best improve the primary goals of medicine [3,4]. There may be plenty of arguments against the representation of all dimensions in PRSs. However, PRSs should not be confused with other well-investigated expert initiatives that measure physician performance and quality of care [6,7,28]. For example, a rather broad representation of dimensions of patients' experience and satisfaction could have a positive impact on the interactive functions of PRSs and thus on the three levels of patients' health literacy [29,30]: Aside from the direct recommendations of individual physicians PRSs bear the potential to improve users' critical reflection about what aspects could and should be considered prior to choosing a doctor. It seems possible, therefore, that PRSs increase the users' health literacy at the *interactive *level, since it is precisely the exchange of information that is considered the essential point of these sites. Concerning the *critical *level of health literacy, one could also expect (in the best case) a similarly positive development. In particular the possibility of evaluating a specific dimension as more or less helpful and the possibility of free narrative commentary could train users to individually filter out what is important - possibly leading the patients to pay more attention to the doctors' competence in shared decision-making and less attention to practices' atmosphere. However, the major precondition for PRSs to achieve these objectives would be to provide possibilities for open narrative commentaries next to structured rating options and to facilitate peer-to-peer communication. Needless to say, PRSs also bear the potential to negatively influence the three levels of health literacy.

Our findings provide the starting point for dealing with another potential impact of PRSs. It also seems plausible to assume that in the future complex constructs such as 'quality of care', 'physician performance', or 'patient satisfaction/experience' will still not be operationalized through consensus on a single gold standard, nor that such a gold standard will be used in all PRSs. Thus the consumer-driven, peer-to-peer operationalization of dimensions such as professional competence and doctor-patient relationship in PRSs could significantly shape the social image of the doctor and the self-understanding on the part of both doctors and patients. An important question in the research accompanying PRSs, therefore, would be: which of the current operationalizations of patients' experiences and satisfaction in general and of professional competence and doctor-patient relation in particular are establishing themselves in PRSs?

Independently of the factual development examined here, the question also arises whether and to what extent health policy can and should influence the operationalization and assessment of patients' experience and satisfaction in consumer-driven PRSs (true Web 2.0 application). One could imagine certifications from professional societies and public institutions entailing not just formal and legal standards but also specifications for a suitable representation and operationalization of patients' experience and satisfaction. Here the concrete difficulty would be to work out a set of dimensions capable of consensus from among the wide range of operationalizations shown by this study. From an ethical viewpoint such certifications would be more legitimate if they arose through a transparent process allowing the participation by various stakeholders (including consumer representatives). This process would have to include discussions of which characteristics of doctors and medical practices are crucial in order to appropriately inform consumers in accordance with the primary goals of medicine (see above).

## Conclusion

The practical relevance of all issues we discussed in the previous paragraphs only increases with a more widespread use of PRSs. At present, the use of PRSs is rather limited. Recent work on the state of PRSs found that reviews were scarce, and when present, most were positive concerning the overall satisfaction with the specific physician [10]. For 300 physicians, only 66 written patient narratives across 33 sites could be identified [10]. However, there are many reasons that argue in favour of a more widespread use of PRSs in the near future. For example, the so-called Facebook or MySpace generation has been especially socialized with the internet. When this generation reaches the age in which they are increasingly interested in health questions and thus doctors, it is likely that the internet will also play a significant role in their decisions (see among other things the rapidly growing number of participants in patient communities [31]).

In the last decade several national health systems have adopted public reporting instruments allowing consumers to make explicit comparisons between the performances of health care providers or health plans in order to make an informed choice [32]. While the aims of public reporting approaches - increasing public accountability, supporting consumer choice and finally improving quality of care - are comparable with the aims of PRSs, they currently lack patient experience and satisfaction information with respect to individual physicians. PRSs might be interesting esspecially for countries that do not follow gatekeeper models and therefore have free physician choice (e.g. Germany).

In light of the mentioned trend towards an increasing practical relevance of PRSs, these new websites bear the potential to influence the public health and health literacy. Future ethical and policy analyses should take these various influences into account and explicitly weigh them before drawing any conclusions on the form and content of PRSs [33].

## Competing interests

The authors declare that they have no competing interests.

## Authors' contributions

SR and DS both contributed to conception, design and acquisition, analysis and interpretation of data. SR and DS drafted and revised the manuscript. Both authors approved the final manuscript.

## Pre-publication history

The pre-publication history for this paper can be accessed here:

http://www.biomedcentral.com/1472-6963/10/332/prepub
